# Therapeutic Benefits of Balneotherapy on Quality of Life of Patients with Rheumatoid Arthritis: A Systematic Review

**DOI:** 10.3390/ijerph182413216

**Published:** 2021-12-15

**Authors:** Maria Fernandez-Gonzalez, Carolina Fernandez-Lao, Lydia Martin-Martin, Angela Gonzalez-Santos, Maria Lopez-Garzon, Lucia Ortiz-Comino, Mario Lozano-Lozano

**Affiliations:** 1 Department of Physical Therapy, Faculty of Health Sciences, University of Granada, 18010 Granada, Spain; mariafg@correo.ugr.es (M.F.-G.); angelagonzalez@ugr.es (A.G.-S.); maloga@ugr.es (M.L.-G.); luciaoc@ugr.es (L.O.-C.); mlozano@ugr.es (M.L.-L.); 2 Instituto de Investigación Biosanitaria ibs.GRANADA, 18016 Granada, Spain; 3 “Cuídate” Support Unit for Oncology Patients, Sport and Health University Research Institute (iMUDS), 18010 Granada, Spain

**Keywords:** balneotherapy, rheumatoid arthritis, quality of life

## Abstract

Background: Rheumatoid arthritis (RA) is the most common inflammatory rheumatic disease. RA symptoms make the disease disabling and strongly impact the quality of life of patients. Among the available forms of treatment, balneotherapy seems to be one of the most common forms of nonpharmacological treatment for rheumatic disease. The aim was to explore the effectiveness of balneotherapy for improving the quality of life of patients with RA. Methods: Pubmed, Scopus, Web of Science and The Cochrane library were searched for randomized or clinical controlled trials published in English or Spanish until May 2021. Risk of bias of included articles were assessed using the Cochrane tool. A total 535 records were retrieved, and seven met the inclusion criteria. All the included studies showed statistically significant improvements in the quality of life of patients who received balneotherapy treatment despite differences in treatment administration. Sessions should be approximately 20 min long and use natural mineral waters enriched with elements, or mud, at a water temperature between 35–38 °C. Conclusions: Balneotherapy benefits the quality of life of people with RA. The obtained results show positive effects for both mineral bathing and immersion in sand or mud on the quality of life of people who suffer from RA.

## 1. Introduction

Rheumatoid arthritis (RA) is one of the most common inflammatory chronic disease [1]. Globally, the proportion of prevalent cases in 2017 was 1.3%, but varies slightly across countries [2]. RA is an autoimmune disease that predominantly affects peripheral joints [3] and may even present extra-articular symptoms [4].

The clinical manifestations of RA depend on the patient, but RA is normally a symmetrical disease that initially affects small joints, such as proximal interphalangeal joints and metacarpophalangeal joints, and then progresses to larger joints [5]. Among the risk factors for RA, 60% are genetic and 40% are environmental factors. Environmental factors include female sex, vitamin D deficit, infectious agents, silica exposure, smoking, obesity and microbiota changes [6]. In RA, the bone and cartilage of affected joints are destroyed, and tendons and ligaments are weakened: this joint damage causes joints deformities and bone erosion that are extremely painful for patients and create an increased risk of falls leading to severe fractures [7,8]. Characteristic symptoms of RA are morning stiffness of the affected joints for more than 30 min, fatigue, tender joints, swollen and warm joints and rheumatoid nodules under the skin. RA has remission and exacerbation periods, and the latter further deteriorate the quality of life (QoL) of patients, making RA an extremely disabling disease [7,9].

Given the chronicity of the disease, there are pharmacological treatments to improve the prognosis of the disease and control the symptoms, although there are now non-pharmacological treatments to reduce the disability caused by RA [6]. One of the most commonly used nonpharmacological treatments for rheumatological disease in European countries is balneotherapy or spa therapy [10]: this therapy is used for osteoarthritis [11], RA [12] or fibromyalgia [13]. Balneotherapy is defined as the use of mineral water, peloids or gases for health promotion and prevention or treatment of diseases. Important features of balneotherapy are the temperature of the mineral water used and the water composition, which depends on the respective country. Mud packs or peloids can be used wet or dry (in the form of sand), and their properties depend on their origin. Waters can be enriched with gases, mainly CO_2_ or radon. All these types of balneotherapy usually take the form of baths for the whole body or the affected portion of the body [14]. The benefits of balneotherapy included improved motion and reduced stiffness and pain. These benefits may have a positive impact on RA symptoms, which could improve the quality of life of patients with RA [14,15,16].

Systematic reviews have been published on the effectiveness of balneotherapy for patients with RA. A review was published in 2002 by Brosseau et al. [12] and another review was published in 2009 by Falaga et al. [17]; however, more than 10 years have passed since the publication of these reviews, which are now out-dated. The Cochrane Organization published a systematic review in 2015 [15] that updated information provided in a systematic review in 2008 on balneotherapy and osteoarthritis [18]. Finally, the latest systematic review was carried out in 2016 by Santos; however, it does not focus on any particular outcome, and new trials have been published [19]. The sparsity of reported scientific evidence on the benefits of balneotherapy is due to none of the aforementioned reviews being focused a specific outcome; instead, several outcomes have been simultaneously explored. Thus, a current update of the scientific evidence is needed to achieve the best response from RA patients. The present systematic review is a compilation of recent results on the benefits or limitations of balneotherapy for the quality of life of patients with RA.

## 2. Materials and Methods

This systematic review has been registered in the PROSPERO International Prospective Register of Systematic Reviews (registration number CRD42021231135). This review was written according to preferred reporting items for systematic review initiatives and meta-analysis [20].

### 2.1. Elegibility

This systematic review includes published articles on RA patients.

The inclusion criteria for the studies were as follows: (a) a methodological design for clinical trials; (b) an eligible population consisting of adults with RA; (c) use of balneotherapy or similar treatment; (d) assessment of the quality of life with any internationally validated instrument; (e) written in English or Spanish; and (f) full-text articles (authors were contacted otherwise).

The exclusion criteria were as follows: (a) any other type of manuscripts such as reviews, guidelines, commentaries and/or case reports, and (b) inclusion of other rheumatic diseases if the results were not analysed by group.

All these inclusion and exclusion criteria were chosen based on the PICO research strategy and the international PRISMA guidelines, following clinical (AR is mainly an adult disease with significant differences in the juvenile population; quality of life is one of the most important variables affected by AR) and methodological criteria (the languages included are those used by the authors, including English, which encompasses the majority of scientific publications; the study design is that which guarantees the best possible scientific evidence).

### 2.2. Outcomes

Changes in the quality of life of patients with RA who received balneotherapy treatment were analysed in this systematic review. Different scales were used: the Health Assessment Questionnaire (HAQ) [21], Visual Analogue Scale (VAS), Arthritis Impact Measurement Scales (AIMS) [22] and patient disease autoevaluation.

### 2.3. Information Sources

Systematic literature searches were conducted using the following electronic databases to identify all relevant studies published up to the search date of May 2021: (a) MEDLINE (via PubMed); (b) Scopus (c) Web of Science; and (d) The Cochrane Library. These four academic and professional databases were chosen in consultation with a medical librarian as being the most appropriate for this review.

### 2.4. Search Strategy

MeSH terms as well as free entry terms (both in English) were included in the search strategy used in this review, combined with Boolean operators (AND, OR), and search techniques, such as truncation, phrase marks or wildcards, adapting them to each database. Terms for different categories were obtained by consulting previous reviews. Appendix A shows the search equations used for the different databases.

### 2.5. Selection Process

**Figure 1:**
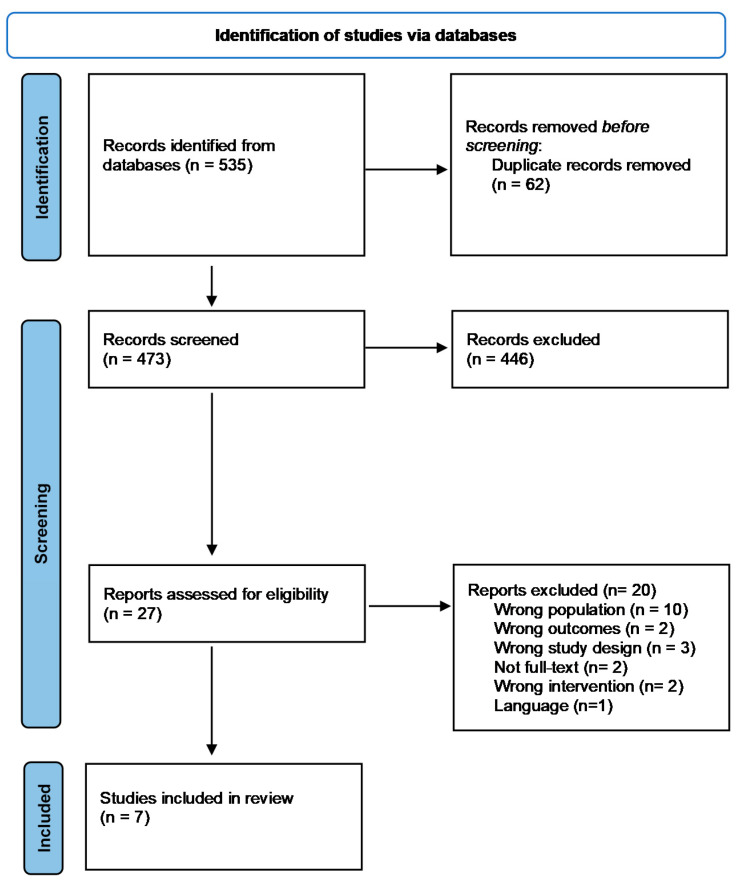
The detailed process of trial selection.

After search results were obtained from all databases, duplicates were removed. Two of the review authors independently selected trials by inspecting titles and abstracts, and irrelevant studies were excluded. In the second screening phase, the complete text of studies was perused, and eligibility criteria were assessed. Disagreements on study eligibility were resolved by external review. Figure 1 shows a PRISMA flow chart for the systematic reviews and meta-analyses [20] used in the selection process. COVIDENCE (Covidence systematic review software, Veritas Health Innovation, Melbourne, Australia, available at www.covidence.org, accessed on 30 July 2021) bibliographic management was used to eliminate duplicates and screen and select the included studies.

Data extraction was performed to assess study quality. To facilitate the process of study selection, extraction and analysis of results, a spreadsheet was designed, including (1) first author, (2) year of publication, (3) design, (4) sample size, (5) intervention, (6) outcomes, and (7) main results.

A narrative synthesis was carried out to comparatively analyse the included studies (a quantitative synthesis was performed based on measures of central tendency, the risk coefficient, 95% confidence intervals and the level of statistical significance of the *p* value, as appropriate). Finally, recommendations for future research or trials were made based on the evidence gathered in this review.

### 2.6. Risk of Bias

Two independent reviewers used the Cochrane Risk of Bias Tool 2 (ROB2) [23] to examine the methodological quality of the included studies, where disagreements were resolved by consensus, and a third reviewer was contacted if consensus was not reached. This tool consists of five domains (a randomisation process, deviations from intended interventions, missing outcome data, measurement of the outcome and selection of the reported results), and the final decisions were low-risk, although there were some concerns or a high risk of bias for the included studies.

## 3. Results

A total of 7 studies were selected for this review. Table 1 shows the main characteristics of the studies in terms of the design, number of participants, intervention used, measures and results. Table 2 shows data extracted from the studies as means and deviation for control and intervention groups with respect to the baseline data for post-treatment and follow-up periods.

### 3.1. Descriptive Study

Most of the studies included in this systematic review were conducted in Europe, specifically in Turkey [27,28], Germany [25,26] and Portugal [29], and compared to studies carried out in Egypt [24] in the African continent and Israel [30] in Asia.

The time distribution of the studies is quite heterogeneous; the earliest studies were published in 1990 [30] and 2000 [26]. The other studies were published recently, in 2013 [25], 2016 [27,29], 2017 [28] and 2018 [24].

All the included studies were randomized controlled trials: in one study, in particular, crossed samples were used throughout the investigation [28].

Similar sample sizes were used in the studies, where the smallest sample size corresponded to a study based on groups of 10 subjects [30]. The largest sample size was approximately 60 patients [26,28], and a sample size of 98 was used in another study [25]. The remaining studies were performed using a sample size of approximately 40 patients [24,27,29]. All the participants in the different trials had RA, with the exception of one trial in which the effects of balneotherapy on different types of rheumatic diseases were evaluated [25]. Furthermore, an inclusion criterion for all trials was a minimum age of 18 years; the mean age of the participants in all the studies was between 40 and 50 years.

### 3.2. Interventions

The studies selected for this review represent different interventions in terms of treatment duration and characteristics.

Two of the selected studies were carried out at the same spa and were therefore performed under similar conditions in terms of the temperature and composition of the mineral water used [27,28]. These mineral waters were rich in sodium chloride and other minerals, such as magnesium and calcium. The bath temperature was approximately 36–37 °C. Two studies were carried out using baths of mineral waters containing mainly carbon dioxide and radon [25,26]. The temperature of these baths oscillated around 36–38 °C. The composition of the water used in for these two studies differed from that of the other studies because the thermal waters were enriched with sulfur [29,30]. In one of these two studies, whole-body sulfur baths at 34 °C with underwater exercises were alternated with sulfur baths at 37 °C with underwater jets directed at painful joints for 10 min [29]. In the second of these two studies, sulfur baths at 37 °C and whole-body mud packs at 42 °C were used [29]. In the last included study, whole-body sand baths were used in conjunction with olive-oil massages [24]. The body was buried in sand at a temperature ranging between 50 and 60 °C.

In all the included studies, each balneotherapy session had the same duration of 20 min. In only one study, one of the two interventions used was performed for 30 min [29].

Similar to the session duration in the studies, very similar numbers of sessions (mostly 12) were used in all the studies. The number of sessions was significantly higher (21) in one study than for the other studies [29]; in other studies, 14 [30] and 15 [26] sessions were performed, and 7 sessions were performed in only one study [24].

The balneotherapy sessions were distributed over time very similarly for all the trials, that is, the sessions which were carried out on consecutive days until the total number of sessions was reached (in some cases, no treatment was administered on Sundays). In the two studies that deviated from this norm, sessions were performed every 2 or 3 days [25,26].

In most of the studies, the intervention group was compared with a control group that only received the usual pharmacological treatment. In two of the included trials, the control group was treated with artificially generated mineral water [25,26]. In one trial, balneotherapy treatment was compared with standard physiotherapy [24], and in another trial, the results of two types of interventions were compared against those of a control group [30].

All patients continued to receive the usual pharmacological treatment in addition to balneotherapy treatment in all the included studies, because pharmacological treatment was a requirement for participating in the studies.

### 3.3. Outcome

The quality of life was the outcome chosen in this systematic review to determine the effectiveness of balneotherapy for patients with RA.

Most of the selected studies used the HAQ scale [21] to evaluate quality of life [24,25,27,28,29]. In four studies, the VAS was used in addition to the HAQ to assess the general condition of the patient [27,28,29]. In one study, the VAS was used as a quality-of-life measurement tool [29].

In one trial, the AIMS [22], which includes questions related to quality of life, was used. In another study, a different tool was used to measure quality of life, that is, the patient’s self-assessment of the disease on a scale from 1 to 10 [30].

### 3.4. Risk of Bias

**Figure 2:**
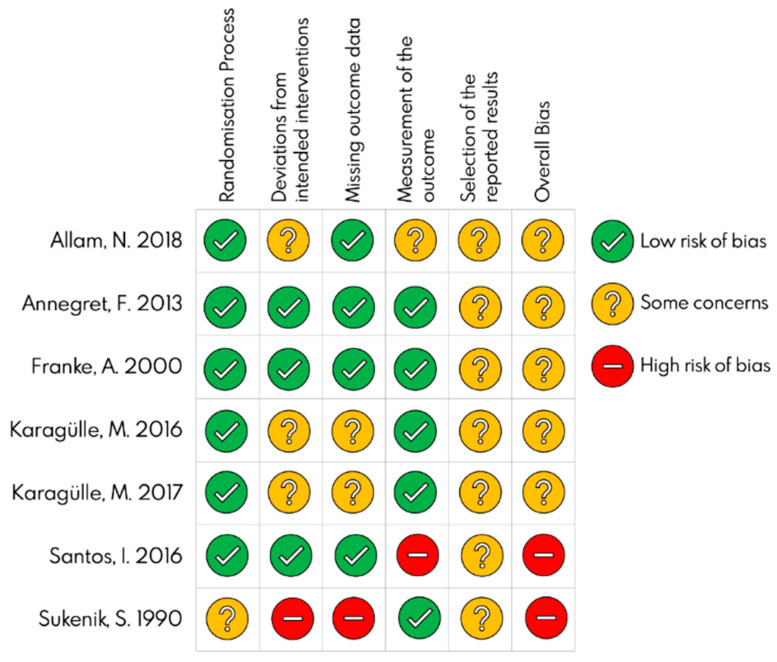
Individual risk of bias [24,25,26,27,28,29,30].

**Figure 3:**
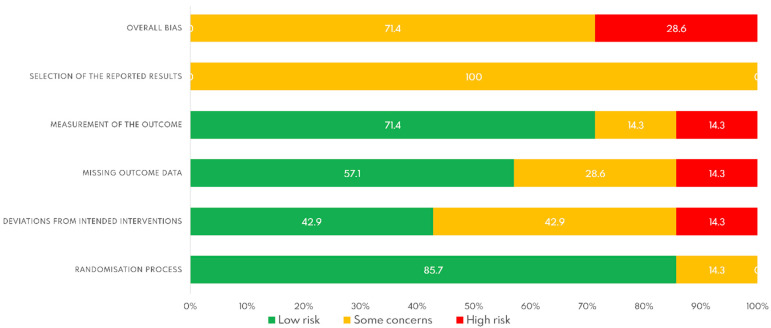
Global risk of bias.

The risk of bias of the included studies was measured using the Cochrane Tool. Figure 2 shows the risk of bias of each study, and Figure 3 shows see the global risk of bias. A high risk of bias for found for the studies by Santos I et al. [29] and Sukenink S et al. [30]. The most repeated bias was the selection of the results because none of the studies included a protocol for the statistical analysis. The next most repeated bias was deviation from the intended intervention.

## 4. Discussion

In this systematic review, the benefits of balneotherapy on the quality of life of patients with RA is evaluated. The obtained results show positive effects for both mineral bathing and immersion in sand or mud on the quality of life of people who suffer from RA. However, in order to analyse the results of the studies included, it is valuable to mention that 3 out of 7 performed a sample size calculation and only two of them showed significant differences between groups [24,26]. The other one did not reach the required sample size [25], so it could be a possible explanation for the lack of significant differences between groups. Additionally, there is probably a lack of statistical power in the significant mean differences showed in Karagülle et al. 2017 and 2018, and Santos et al. 2016 due to the fact that none of them calculated sample size before intervention. Therefore, their results could be inconclusive [27,28].

Differences in treatment administration resulted from the water composition (due to natural enrichment with radon or carbon dioxide) and whether water baths were administered in conjunction with pressure jets or sand baths. Similar bath temperatures were used in all the studies and ranged between 35 and 38 °C. The session duration was homogeneous across studies at approximately 20 min. Statistically significant results were found in all studies despite the treatment differences. This result may have been obtained because the salient feature of balneotherapy is not the mineral enriching the water, but rather that the water itself is natural, warm and produces a state of wellness that in turn leads to a body response that improves both quality of life and pain, as has been reported in previous reviews [17].

There were differences in administration protocols for balneotherapy treatment among the studies, where a few sessions were used in some studies and treatment was administered for 21 days in others; however, statistically significant results were obtained in all studies with respect to the quality of life of patients with RA. Similar results have been obtained in studies on the quality of life of the osteoarthritis population, as shown by the review published by Verhagen et al. [18]. However, Kamioka et al. [31] published a review in 2010 showing that no conclusions could be drawn on the efficacy of balneotherapy in RA. We propose that this inference may have resulted from the low methodological quality of the studies published on using balneotherapy to treat RA, which demonstrates the scarcity of publications on this subject.

The instruments used to assess the main variable of this systematic review were highly heterogeneous. The most commonly used questionnaire is the HAQ [24,25,27,28,29]. All evaluations were performed post-intervention, except that Karagülle et al. [28] evaluated the quality of life at 3 months after treatment and at 6 months. These evaluations were statistically significant for all studies post-intervention but not after 6 months. Short-term results show that balneotherapy benefits the quality of life of patients, but these positive effects may not endure over time. Studies on the long-term effects of balneotherapy for patients with RA have not yet been carried out; thus, a systematic review should be carried out in the future to gather new evidence on the effectiveness of balneotherapy treatment several months after the intervention is performed.

Finally, the effects of thermal baths are partially related to temperature. Hot stimuli may influence muscle tone and pain intensity, helping to reduce muscle spasm and to increase the pain threshold in nerve endings. According to the “gate theory”, pain relief may be due to the temperature and hydrostatic pressure of water on the skin. Thermal stress provokes a series of neuroendocrine reactions. In particular, the heat stimulates the release of adrenocorticotropic hormone (ACTH), cortisol, prolactin and growth hormone (GH), although it does not alter the circadian rhythm of these hormones. The effect of thermal stress on the hypothalamus-pituitary-adrenal axis seems to be particularly important for the antiedemigenous and anti-inflammatory actions of corticosteroids, as well as for the frequent alteration of the axis during some RDs28. The increase in beta-endorphin demonstrated to occur with various spa therapy techniques has an analgesic and anti-spastic effect that is particularly important in patients for whom pain is the prevalent symptom, which will ultimately influence their quality of life [32]. Furthermore, there seems to be evidence of the relationship and positive effects of balneotherapy in limiting the production of pro-inflammatory cytokines, prostaglandins or even adipokines in patients with RA [33]. It seems that balneotherapy has a capacity to regulate and reduce the parameters of oxidative status in these patients [34] and, in addition, it seems that it may also have an effect in reducing the main markers of bone and cartilage damage [35]. All this, together with the positive clinical results presented in this review, highlights the need to include this therapy as part of the non-pharmacological intervention for these patients.

### 4.1. Limitations and Strenghts

This systematic review is not without limitations. The heterogeneity in evaluating the quality of life of patients with RA makes it difficult to study this topic in depth and prevents quantitatively combining the results of the included studies. In fact, as reflected in our initial PROSPERO registry, we initially intended to include studies that measured quality of life using SF-36 or AIMS tools (AIMS was only used in one study [26] included in this review); however, as other tools were used in studies of interest in the field, we changed the inclusion criterion and finally included studies in which other tools, such as HAQ or VAS, were used to evaluate quality of life. Another limitation is the lack of studies in languages other than English or Spanish: some studies that met the inclusion criteria a priori were not included in this review, because although the abstracts were in English, the complete text was only available in German or Japanese. Finally, no specific age criterion was set for inclusion criteria, leaving it open to adults with RA.

A strength of this review is the inclusion of only randomized controlled trials, which are studies based on scientific evidence. Thus, all studies that scientifically evaluated the quality of life were considered, regardless of the measurement tool used. The progress of science depends on the development of versions where there is consensus on the current scientific evidence. This consensus is created, among others, by the development of methodologically sound systematic reviews, to clarify the state of knowledge and identify starting points for future research. Reviews provide, at the very least, a quick way to become familiar with the topic and the main findings on the subject, as well as providing useful syntheses of the research findings [36]. Our purpose was writing a readable synthesis of the best resources available in the literature for this area of research. Our review methods have been critical because we provided an unbiased point of view for the reader regarding the current topic.

### 4.2. Clinical Implications

Six [24,25,27,28,29,30] of the seven included studies show statistically significant intra-group differences in the intervention group (seventh shows inter-group differences [26]), as well as five [24,26,27,28,29] of the six studies reporting inter-group data show statistically significant differences in favour of the experimental group. Therefore, it appears that the combination of balneotherapy with pharmacological treatment shows improvements in the quality of life of these patients compared to receiving only pharmacological treatment, receiving standard physical therapy or receiving artificially generated mineral water in balneotherapy treatment. Despite the low methodological quality of studies in this field, we were able to use the results of the studies included in this review to design an effective balneotherapy treatment, although a field study to corroborate this design has not been performed. Based on the results of this systematic review, sessions should be approximately 20 min long and use natural mineral waters enriched with an element, such as radon, or carbon dioxide. Each session should consist of whole-body immersion in these waters, without moving the body or performing any type of exercises, at a water temperature between 35–38 °C. Hot sand baths could be used instead of mineralised waters, or even a combination thereof, as both water and sand have beneficial effects. The total number of sessions should be approximately 15, and the sessions should be performed on consecutive days. The maintenance of pharmacological treatment is essential for balneotherapy treatment to be effective.

## 5. Conclusions

Balneotherapy seems to improve the quality of life of patients with RA. This is due to the results showed in studies, because in all of them the scores regarding quality of life are improved after balneotherapy treatment. Despite being beneficial, balneotherapy continues to be investigated. Therefore, it is necessary to carry out field investigations to clarify the uncertainties that still exist regarding treatment efficacy, such as determining the type of mineral water that is most beneficial for RA, not only in terms of quality of life, but also regarding pain or functionality. Additionally, research is needed to determine the best form of balneotherapy for patients with RA, by selecting between baths of mineral waters versus mud or sand. Based on the results of this systematic review, which point to the benefits of this technique in this population, as well as the few RCTs and their methodological analysis, there is a critical need to develop more RCTs with a larger sample size and to overcome some of the biases detected, analysing in depth the benefits of this technique in rheumatoid arthritis.

## Figures and Tables

**Table 1:** Main characteristic of selected studies.

Author, Year	Desing	Participant	Intervention	Scales	Secondary Outcomes	Results
Allam, N.M., et al. [24] 2018	RCT	N = 30 IG; *n* = 15 CG; *n* = 15	IG: Siwan therapy 7 days of hot sand baths for 20 min and massage with olive oil CG: standard physiotherapy treatment	HAQ	Pain (VAS)	The scale score were decreased significantly compared with baseline in spa group but not in control group
Annegret, F., et al. [25] 2013	RCT	N = 98 IG; *n* = 48 CG; *n* = 50	3–4 weeks of intervetion IG: 12 total radon baths every 2 or 3 days in spedificil locations for 20 min a day CG: 12 tap water baths with artificial CO_2_ for 20 min	HAQ	Pain (VAS) SF-12	There is a significant improvement in the HAQ scores of the intervetion group versus the control group
Annegret, F., et al. [26] 2000	RCT	N = 60 IG; *n* = 30 CG; *n* = 30	4 weeks of intervetion IG: 15 total radon baths for 20 min a day CG: 15 baths in tap water baths with artificial CO_2_ for 20 min	AIMS	Pain (VAS) Keitel funcional test (KFI)	Significant differences were found in terms of the improvement in the result of the AIMS scale in the intervention group at 3 and 6 months of follow-up.
Karagülle, M., et al. [27] 2017	RCT	N = 37 IG; *n* = 15 CG; *n* = 22	2 weeks of intervetion IG: 12 balneotherapy sessions in mineral water for 20 min a day CG: standard drug treatment	HAQ	Pain (VAS) Disease Activity Score (DAS28) Patient global assessment (VAS) Biochemical analysis	HAQ scores were significantly reduced compared to baseline results in the intervention group, but not in the control group
Karagülle, M., et al. [28] 2018	CRCT	N first period = 15 N second period = 22	2 weeks of intervetion IG: 12 balneotherapy sessions in mineral water for 20 min a day CG: standard drug treatment	HAQ	Pain (VAS) Disease Activity Score (DAS28) Patient global assessment (VAS)	A significant difference was found in the intervention group at 3 months; however, there were no significant differences at 6 months
Santos, I., et al. [29] 2016	RCT	N = 44 IG; *n* = 22 CG; *n* = 22	21 days of intervention IG: two treatments on alternate days. Treatment 1 consisted of sulphur baths of 30 min and underwater exercises. Treatment 2 consisted of sulphur baths of 20 min and underwater jets for 10 min in painful joints CG: standard drug treatment	HAQ	Global health assessment (VAS) Pain (VAS) Quality of life (VAS) Fatigue (VAS) Disease Activity Score (DAS28)	HAQ improved significantly in the intervention group compared to the control group
Sukenik, S., et al. [30] 1990	RCT	N = 40 4 groups of 10 patiens each one	2 weeks fo intervetion Group 1: daily mud packs in full body for 20 min a day Group 2: daily sulphur baths for 20 min a day Group 3: combination of daily mud packs and daily sulphur baths Group 4: control, without treatment	Patient assessment of disease severity	Morning stiffness Fifteen metre walk time Circunference of proximal interphalangeal joints Activities of daily living	Significant improvements were found in terms of the patient’s perception of the disease in the three treatment groups

Note: RCT = randomized control trial; CRCT = crossover randomized controlled trial; N = sample size; IG = intervention group; CG = control group; HAQ = health assessment questionnaire; VAS = visual analogue scale.

**Table 2:** Data of the included studies.

Author, year	Scales	Baseline Evaluation		Post-Treatment Evaluation				Follow-Up			
		Control Group	Intervention Group	Control Group	Intervetion Group	Intragroup	Intergroup	Control Group	Intervention Group	Intragroup	Intergroup
Allam, N.M., et al. [24] 2018	HAQ	1.82 ± 0.52	1.75 ± 0.58	1.58 ± 0.67	0.82 ± 0.50	*p* < 0.05 in intervention group	*p* < 0.05 in favor to intervention group	-	-	-	-
Annegret, F., et al. [25] 2013 ^a^	HAQ	0.95 (0.62)	0.93 (0.52)	0.10 (0.29)	0.08 (0.39)	*p* < 0.05 in intervention group	*p* > 0.05	3 months = 0.08(0.31) 6 months = 0.07(0.43) 9 months = 0.07(0.34)	3 months = 0.10 (0.42) 6 months = 0.17(0.37) 9 months = 0.09 (0.45)	*p* > 0.05	*p* < 0.05
Annegret, F., et al. [26] 2000 ^a^	AIMS	6.60 (1.10)	6.27 (1.33)	-	-	*p* > 0.05	*p* < 0.05 in favor to intervention group	3 months = −0.06 (−0.34, 0.23) 6 months = −0.18 (−0.36, 0.20)	3 months = 0.41 (0.06, 0.75) 6 months = 0.41 (0.06, 0.74)		
Karagülle, M., et al. [27] 2017	HAQ	1.43 ± 0.76	1.33 ± 0.68	2 weeks post tto. 1.23 ± 0.75	2 weeks post tto. 0.79 ± 0.64	*p* < 0.05 in intervention group	*p* < 0.05 in favor to intervention group	-	-		
Karagülle, M., et al. [28] 2018 ^a^	HAQ	0.96 (0.66, 1.99)	1.40 (0.73, 1.83)	2 weeks post tto. 1.10 (1.51, 1.76)	2 weeks post tto. 0.08 (0.33, 1.13)	*p* < 0.05 in intervention group	*p* < 0.05 in favor to intervention group	3 months = 1.00 (0.51, 1.76) 6 months = 1.10 (0.41, 1.55)	3 months = 0.60 (0.40, 0.98) 6 months = 0.65 (0.38, 1.43)		
Santos, I., et al. [29] 2016 ^a^	HAQ	1.34 (0.97, 1.7)	1.50 (1.24, 1.76)	Difference between groups 0.37 (0.09, 0.64)		-	*p* < 0.05	Difference between groups at 3 months 0.44 (0.15, 0.72)			*p* < 0.05
Sukenik, S., et al. [30] 1990 ^b^	Patient’s self assessment of the disease	5.5	Mud packs 4.1	Mud packs 6.5	6.1	*p* < 0.05 in the three treatment groups		3 months Mud packs 5.7	3 months 5.7		
			Sulphur baths 5.0	Sulphur baths 6.4				3 months Sulphur baths 6.3			
			Combination 4.8	Combination 7.0				3 months Combination 5.7			

Note: ^a^ mean change at 95% confidence; ^b^ mean.

## Appendix A. Search Strategy

### Appendix A.1. Pubmed

((((((((((((((((((((((((((((((((((balneology[MeSH Terms]) OR (ammotherapy[MeSH Terms])) OR (baths[MeSH Terms])) OR (mud therapy[MeSH Terms])) OR (steam bath[MeSH Terms])) OR (balneology[Title/Abstract])) OR (ammotherapy[Title/Abstract])) OR (baths[Title/Abstract])) OR (“mud therapy”[Title/Abstract])) OR (“steam bath”[Title/Abstract])) OR (Balneotherapy[Title/Abstract])) OR (“natural mineral water”[Title/Abstract])) OR (spa[Title/Abstract])) OR (“health spa”[Title/Abstract])) OR (“health spas”[Title/Abstract])) OR (“Wellness Centers”[Title/Abstract])) OR (“Wellness Center”[Title/Abstract])) OR (“Wellness Centre”[Title/Abstract])) OR (“Sand Bath”[Title/Abstract])) OR (“Sand Baths”[Title/Abstract])) OR (Sandbath[Title/Abstract])) OR (Sandbaths[Title/Abstract])) OR (Fangotherapy[Title/Abstract])) OR (“Mud Pack”[Title/Abstract])) OR (“Mud Packs”[Title/Abstract])) OR (“Peat Therapy”[Title/Abstract])) OR (“Peloid Therapy”[Title/Abstract])) OR (“Mud Bath”[Title/Abstract])) OR (“Mud Baths”[Title/Abstract])) OR (“Sweat Lodge”[Title/Abstract])) OR (“Finnish Bath”[Title/Abstract])) OR (Sauna[Title/Abstract])) OR (“Finnish Sauna”[Title/Abstract])) OR (“Spa therapy”[Title/Abstract])) OR (“spa therapies”[Title/Abstract])

AND

((((arthritis[MeSH Terms]) OR (arthritis, rheumatoid[MeSH Terms])) OR (“rheumatoid arthritis”[Title/Abstract])) OR (arthritis[Title/Abstract])) OR (rheumatoid [Title/Abstract])

AND

(((((((((((((((“randomized controlled clinical trial”[Title/Abstract]) OR (“randomised controlled clinical trial”[Title/Abstract])) OR (“randomized controlled trial”[Publication Type])) OR (controlled clinical trials, randomized[MeSH Terms])) OR (clinical trial[MeSH Terms])) OR (randomized controlled trial[MeSH Terms])) OR (randomized controlled trials as topic[MeSH Terms])) OR (“randomized controlled trial”[Title/Abstract])) OR (“randomised controlled trial”[Title/Abstract])) OR (“clinical controlled trial”[Title/Abstract])) OR (“controlled clinical trial”[Title/Abstract])) OR (“clinical trial”[Title/Abstract])) OR (“random allocation”[Title/Abstract])) OR (“randomly allocated”[Title/Abstract])) OR (“comparative study”[Title/Abstract])) OR (comparative study[Publication Type])

AND

“Quality of Life”[Mesh Terms] OR (“Quality of Life”[Title/Abstract]) OR (QOL[Title/Abstract]) OR (“Health Related Quality Of Life”[Title/Abstract]) OR (“Health-Related Quality Of Life”[Title/Abstract]) OR (HRQOL[Title/Abstract]) OR (“Life quality”[Title/Abstract])

### Appendix A.2. Scopus

((TITLE-ABS-KEY (balneology) OR TITLE-ABS-KEY (ammotherapy) OR TITLE-ABS-KEY (baths) OR TITLE-ABS-KEY (“mud therapy”) OR TITLE-ABS-KEY (“steam bath”) OR TITLE-ABS-KEY (“natural mineral water”) OR TITLE-ABS-KEY (spa) OR TITLE-ABS-KEY (“health spa”) OR TITLE-ABS-KEY (“wellness center”) OR TITLE-ABS-KEY (“wellness centers”) OR TITLE-ABS-KEY (“wellness centre”) OR TITLE-ABS-KEY (“wellness centres”) OR TITLE-ABS-KEY (“sand bath”) OR TITLE-ABS-KEY (“sand baths”) OR TITLE-ABS-KEY (sandbath) OR TITLE-ABS-KEY (sandbaths) OR TITLE-ABS-KEY (fangotherapy) OR TITLE-ABS-KEY (“mud pack”) OR TITLE-ABS-KEY (“mud packs”) OR TITLE-ABS-KEY (“peat therapy”) OR TITLE-ABS-KEY (“peloid therapy”) OR TITLE-ABS-KEY (“mud bath”) OR TITLE-ABS-KEY (“mud baths”) OR TITLE-ABS-KEY (“sweat lodge”) OR TITLE-ABS-KEY (“finnish bath”) OR TITLE-ABS-KEY (sauna) OR TITLE-ABS-KEY (“finnish sauna”) OR TITLE-ABS-KEY (“spa therapies”) OR TITLE-ABS-KEY (“spa therapy”))) AND ((TITLE-ABS-KEY (“arthritis rheumatoid”) OR TITLE-ABS-KEY (“rheumatoid arthritis”) OR TITLE-ABS-KEY (arthritis) OR TITLE-ABS-KEY (rheumatoid))) AND ((TITLE-ABS-KEY (“Randomised controlled clinical trial”) OR TITLE-ABS-KEY (“Randomized Controlled clinical Trial”) OR TITLE-ABS-KEY (“randomized controlled trial”) OR TITLE-ABS-KEY (“randomised controlled trial”) OR TITLE-ABS-KEY (“controlled clinical trials ranzomized”) OR TITLE-ABS-KEY (“clinical trial”) OR TITLE-ABS-KEY (“controlled clinical trial”) OR TITLE-ABS-KEY (“clinical controlled trial”) OR TITLE-ABS-KEY (“random allocation”) OR TITLE-ABS-KEY (“randomly allocated”) OR TITLE-ABS-KEY (“comparative study”))) AND ((TITLE-ABS-KEY (“Quality of Life”) OR TITLE-ABS-KEY (qol) OR TITLE-ABS-KEY (“Health Related Quality of Life”) OR TITLE-ABS-KEY (“Health-Related Quality Of Life”) OR TITLE-ABS-KEY (hrqol) OR TITLE-ABS-KEY (“Life quality”)))

### Appendix A.3. Web of Sciencie

(“balneology” OR “ammotherapy” OR “baths” OR “mud therapy” OR “steam bath” OR “natural mineral water” OR “spa” OR “health spa” OR “wellness center” OR “wellness centers” OR “wellness centre” OR “wellness centres” OR “sand bath” OR “sand baths” OR “sandbath” OR “sandbaths” OR “fangotherapy” OR “mud pack” OR “mud packs” OR “sandbath” OR “peat therapy” OR “peloid therapy” OR “mud bath” OR “mud baths” OR “sweat lodge” OR “finnish bath” OR “sauna” OR “finnish sauna” OR “spa therapies” OR “spa therapy”)

AND

(“arthritis rheumatoid” OR “rheumatoid arthritis” OR “arthritis” OR “rheumatoid”)

AND

(“Randomised controlled clinical trial” OR “Randomized Controlled clinical Trial” OR “randomized controlled trial” OR “randomised controlled trial” OR “controlled clinical trials ranzomized “ OR “clinical trial” OR “controlled clinical trial” OR “clinical controlled trial” OR “random allocation” OR “randomly allocated” OR “comparative study”)

AND

(“Quality of Life” OR “QOL” OR “Health Related Quality Of Life” OR “Health-Related Quality Of Life” OR “HRQOL” OR “Life quality”)

### Appendix A.4. Cochrane Library

“balneology” OR “ammotherapy” OR “baths” OR “mud therapy” OR “steam bath” OR “natural mineral water” OR “spa” OR “health spa” OR “wellness center” OR “wellness centers” OR “wellness centre” OR “wellness centres” OR “sand bath” OR “sand baths” OR “sandbath” OR “sandbaths” OR “fangotherapy” OR “mud pack” OR “mud packs” OR “sandbath” OR “peat therapy” OR “peloid therapy” OR “mud bath” OR “mud baths” OR “sweat lodge” OR “finnish bath” OR “sauna” OR “finnish sauna” OR “spa therapies” OR “spa therapy” in Title Abstract Keyword

AND

“arthritis rheumatoid” OR “rheumatoid arthritis” OR “arthritis” OR “rheumatoid” in Title Abstract Keyword

AND

“randomised controlled clinical trial” OR “Randomized Controlled clinical Trial” OR “randomized controlled trial” OR “randomised controlled trial” OR “controlled clinical trials ranzomized “ OR “clinical trial” OR “controlled clinical trial” OR “clinical controlled trial” OR “random allocation” OR “randomly allocated” OR “comparative study” in Title Abstract Keyword-(Word variations have been searched)

AND

“Quality of Life” OR “QOL” OR “Health Related Quality Of Life” OR “Health-Related Quality Of Life” OR “HRQOL” OR “Life quality” in Title Abstract Keyword
